# Renal thrombotic microangiopathy in patients with cblC defect: review of an under-recognized entity

**DOI:** 10.1007/s00467-016-3399-0

**Published:** 2016-06-11

**Authors:** Bodo B. Beck, FrancJan van Spronsen, Arjan Diepstra, Rolf M. F. Berger, Martin Kömhoff

**Affiliations:** 1 Institute of Human Genetics, University of Cologne, Cologne, Germany; 2 Division of Metabolic Diseases, Beatrix Children’s Hospital, University Medical Centre Groningen, University of Groningen, Groningen, The Netherlands; 3 Department of Pathology and Medical Biology, University Medical Centre Groningen, University of Groningen, Groningen, The Netherlands; 4 Centre for Congenital Heart Diseases-Paediatric Cardiology, University Medical Centre Groningen, University of Groningen, Groningen, The Netherlands; 5 Division of Paediatric Nephrology, Beatrix Children’s Hospital, University Medical Centre Groningen, University of Groningen, Groningen, The Netherlands; 6 University Children’s Hospital, Philipps University Marburg, Baldinger Str., 35043 Marburg, Germany

**Keywords:** Atypical hemolytic uremic syndrome, Cobalamin C defect, Pulmonary arterial hypertension, Children

## Abstract

Methylmalonic aciduria and homocystinuria, cobalamin C (cblC) type, is the most common genetic type of functional cobalamin (vitamin B_12_) deficiency. This metabolic disease is characterized by marked heterogeneity of neurocognitive disease (microcephaly, seizures, developmental delay, ataxia, hypotonia) and variable extracentral nervous system involvement (failure to thrive, cardiovascular, renal, ocular) manifesting predominantly early in life, sometimes during gestation. To enhance awareness and understanding of renal disease associated with cblC defect, we studied biochemical, genetic, clinical, and histopathological data from 36 patients. Consistent clinical chemistry features of renal disease were intravascular hemolysis, hematuria, and proteinuria in all patients, with nephrotic-range proteinuria observed in three. Renal function ranged from normal to renal failure, with eight patients requiring (intermittent) dialysis. Two thirds were diagnosed with atypical (diarrhea-negative) hemolytic uremic syndrome (HUS). Renal histopathology analyses of biopsy samples from 16 patients revealed glomerular lesions typical of thrombotic microangiopathy (TMA). Treatment with hydroxycobalamin improved renal function in the majority, including three in whom dialysis could be withdrawn. Neurological sequelae were observed in 44 % and cardiopulmonary involvement in 39 % of patients, with half of the latter group demonstrating pulmonary hypertension. Mortality reached 100 % in untreated patients and 79 and 56 % in those with cardiopulmonary or neurological involvement, respectively. In all patients presenting with unclear intravascular hemolysis, hematuria, and proteinuria, cblC defect should be ruled out by determination of blood/plasma homocysteine levels and/or genetic testing, irrespective of actual renal function and neurological status, to ensure timely diagnosis and treatment.

**Electronic supplementary material:**

The online version of this article (doi:10.1007/s00467-016-3399-0) contains supplementary material, which is available to authorized users.

## Introduction

Vitamin B_12_, also known as cobalamin (Cbl), is a complex water-soluble organic compound that is essential to all animals and numerous microorganisms. From an evolutionary perspective, vitamin B_12_ is an extremely old molecule, which has even been suggested to be synthesized prebiotically [1]. Synthesis is limited to several archaea and eubacteria and requires more than 25 steps [2]. The standard Western diet contains 5–7 µg of Cbl per day, which comes exclusively from dairy products and meat. The liver stores sufficient Cbl for several years, even after intake has completely ceased. The absorption, transport, storage, and intracellular processing of Cbl is complex, as is reflected by the high number of at least 21 genes that are known to affect Cbl absorption, storage, and metabolism in humans [3]. Vitamin B_12_ deficiency can thus result from numerous primary (genetic) and secondary conditions.

**Fig. 1:**
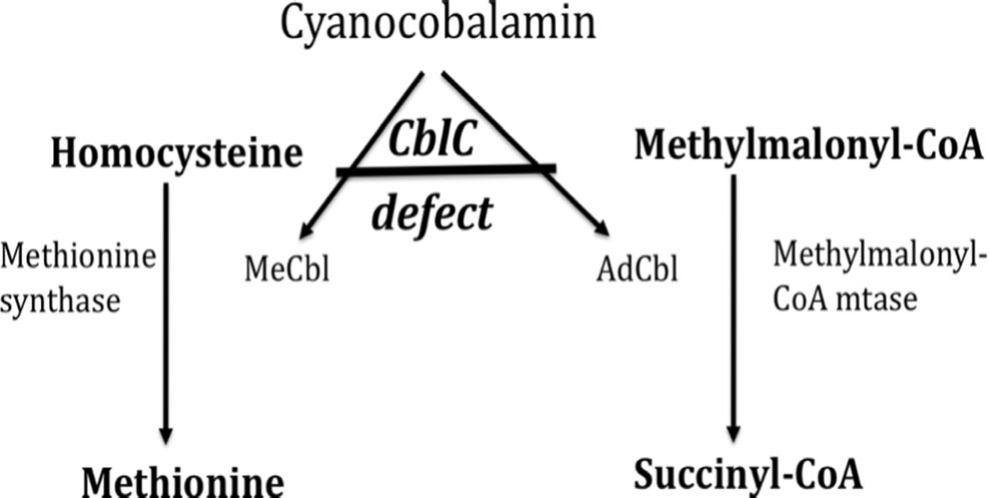
Methylmalonic aciduria and homocystinuria, cobalamin C (cblC) complementation type (MMACHC) is required for decyanation of cyanocobalamin, a precursor for subsequent conversion into the essential cofactors methylcobalamin (MeCbl) and adenosylcobalamin (AdoCbl). MeCbl is required to metabolize homocysteine to methionine, and AdoCbl is needed for the breakdown of methylmalonic acid. This scheme illustrates why cyanocobalamin is largely ineffective to treat cblC defect and why blood levels of both homocysteine and methylmalonic acid are increased while methionine concentrations are reduced (modified from [7], used with permission)

The most common disorder of intracellular vitamin B_12_ metabolism is methylmalonic aciduria and homocystinuria, cblC complementation type (MMACHC; phenotype MIM# 277400), which accounts for ~80 % of all cases. MMACHC has also been designated as cblC defect, cobalamin deficiency, C type, or cobalamin C deficiency. The last two terms could be confused with mere Cbl deficiency/absence (i.e., nutritional Cbl/B_12_ deficiency). To avoid erroneous exclusion of MMACHC based on the observation of normal or even elevated plasma B_12_ levels typically seen in affected individuals, the designations MMACHC or cblC defect is used throughout this review for the autosomal recessive metabolic disorder [4]. MMACHC was first described clinically in 1969 in a child with severe neurological compromise, hyperhomocysteinemia (−uria) with low methionine concentrations, and methylmalonic acidemia/aciduria, who died at 8 weeks of age [5]. Patients with MMACHC show downstream cytoplasmic blocking of Cbl conversion, resulting in combined deficiency of two important cofactors: adenosylcobalamin (AdoCbl) and methylcobalamin (MeCbl) [6]. MeCbl is the coenzyme for methionine synthase (MTR; defective in autosomal recessive cblG complementation type) required for the conversion of homocysteine to methionine in the cytoplasm. When this reaction is impaired, deranged folate metabolism causes—via defective DNA synthesis—the megaloblastic maturation pattern observed in these patients. AdoCbl is the cofactor of the mitochondrial enzyme methylmalonyl coenzyme A (CoA) mutase (itself defective in autosomal recessive methylmalonic acidemia, cblA, or cblB complementation type caused by mutations in either the *MMAA* or *MMAB* gene), which converts L-methylmalonyl-CoA to succinyl-CoA. In the absence of AdoCbl, methylmalonic acid is generated from excess of L-methylmalonyl-CoA (Fig. 1).

This explains the biochemical hallmarks of MMACHC (Table 1): hyperhomocysteinemia/hyperhomocystinuria with low methioninemia, and methylmalonic acidemia/aciduria (which is also the biochemical phenotype seen in the rarer complementation types cblD, cblF, cblJ, and cblX variants [3]).

**Table 1:** Key laboratory parameters in cobalamin C (cblC) defect

Test	Normal range	Patients
Plasma tHCY (μm)	5–15	23–186
Plasma MMA (μm)	<0.27	1.6–14.5
Plasma B_12_ levels (pg/ml)	170–800	611–932

*tHCY* total homocysteine, *MMA* methylmalonic acid

The clinical findings are summarized in Table 2 and are described in detail in a recent review [4].

**Table 2:** Clinical synopsis of myriad methylmalonic aciduria and homocystinuria, cobalamin C (cblC) complementation type (MMACHC) manifestations (modified from [8], used with permission)

System	Clinical manifestations
Growth and habitus	Prenatal growth retardation
	Postnatal failure to thrive
	Microcephaly
	Hydrops fetalis
	Hydrocephalus
	Marfanoid habitus
	Dysmorphic facial features
Central nervous system	Developmental delay
	Seizures
	Ataxia
	Hypotonia
	Lethargy, progressive encephalopathy
	Regression, dementia
	Cognitive impairment ranging from executive dysfunction to severe mental retardation
	Neuropsychiatric disturbances
	Subdural hematoma
	Demyelinating neuropathy Leukoencephalopathy Basal ganglia lesions (less frequent)
Eye	Maculopathy
	Retinal degeneration
	Optic atrophy
	Nystagmus
Blood	Anemia, thrombocytopenia and/or neutropenia, megaloblastosis
Vascular	Recurrent venous thrombosis
	Cor pulmonale or subclinical pulmonary thrombosis
	Cerebrovascular complications, stroke
Renal	Hemolytic-uremic syndrome
	Chronic thrombotic microangiopathy Nephrotic syndrome Renal failure
Heart	Fetal dilated cardiomyopathy Congenital heart defects
	Pulmonary arterial hypertension Left ventricular noncompaction

The gene *MMACHC* (MIM# 609831) was identified in 2006 [9] and is located on chromosome 1p34 and contains only four coding exons. The complementary DNA (cDNA) has an 846-bp open reading frame encoding a protein of 282 amino acids with a predicted molecular weight of 31.7 kD. Contrary to the N-terminal domain of the protein (MMACHC), which lacks homology to any known protein, homology exists between the C-terminal domain and the bacterial protein TonB [9], a Cbl-binding protein [10].

The MMACHC protein (Uniprot KB entry Q9Y4U1) possesses decyanase, dealkylase, and flavin reductase activities. Decyanation of cyanocobalamin is essential for its subsequent conversion into the active cofactors adenosylcobalamin and methylcobalamin (Fig. 1) [11]. This explains why MMACHC patients do not respond to therapy with orally administered cyanocobalamin and should be treated with hydroxycobalamin i.m., which can be metabolized into adenosylcobalamin and methylcobalamin. MMACHC knockout mice die from pre-implantation defect [12]. During midgestation in murine development, MMACHC is expressed in many tissues, including the mesonephric mesenchyme of the kidney and the endothelium of blood vessels and heart [13, 14]. MMACHC represents the prototypic and most common disorder of intracellular Cbl metabolism; currently, 81 different mutations [Human Gene Mutation Database (HGMD) professional 2015.4), and well over 500 patients have been reported worldwide [9, 15–17]. The frameshift mutation c.271dupA (p.R91KfsX14) accounts for about 40 % of disease-causing alleles in the Caucasian population [15], while c.609G>A (p.R161X) has been identified as the most frequent mutation in a study from China [16]. Early-onset disease, defined as manifestation within the first year of life, has been associated with the truncating alleles c.271dupA or c.331C>T (p.R111X) [15]. Two recent studies found incidence rates ranging from 1:100,000 births in the New York, USA, region [13] to 1: 60,000 in California, USA and up to 1:37,000 in the Hispanic population. French Canadian, Cajun, Indian, Chinese, Middle Eastern, and European populations are believed to be at an increased risk for cblC type [8].

Although MMACHC constitutes a multisystemic disease that can affect almost any organ system, including the kidney (see overview in Table 2), cblC defect is largely regarded as a disease affecting central nervous system (CNS) development and neurocognition [18]. In 1979, Baumgartner and her colleagues described the first patient with renal disease and MMACHC defect [19]. The boy from unrelated Italian parents presented in the neonatal phase with failure to thrive and anemia. At 3 months of age, he was diagnosed with elevated methylmalonic acid and homocysteine concentrations in blood and urine. Treatment with cyanocobalamin was unsuccessful. Severe metabolic acidosis and uremia were noted. He developed progressive arterial hypertension, hepatomegaly, and tachypnea. On chest X-ray, cardiomegaly and pulmonary edema were noted. Digoxin and furosemide led to some improvement, but at the age of 4 months, he had a second episode of heart failure and died a few hours later from intractable pulmonary edema. From the first report until to date, at least 36 patients with cblC defect and thrombotic microangiopathy (TMA) have been identified worldwide. The purpose of this review is to increase awareness of the renal manifestation and outcome in MMACHC. 

MMACHC as a treatable form of TMA has received comparatively little attention in the field, as is reflected by its omission in several reviews on TMAs. This is very unfortunate, as the mortality rate of patients with MMACHC is high, while initial screening for elevated homocysteine plasma/blood levels is widely available, fast, and inexpensive (~US $30). Unlike in other rare kidney diseases, a causal therapeutic option, hydroxycobalamin, is readily available. Treatment with hydroxycobalamin is effective in many patients when started early, it has minimal side effects, and it is available at negligible costs (US $1/day).

## Methods

We reviewed clinical data from patients referred to our institutions as well as from published case reports and case series of renal disease in patients with MMACHC. The supplemental table lists biochemical data and provides clinical information of patients with MMACHC for whom individual clinical details of renal disease were available. PubMed search terms included cblC, cobalamin, MMACHC, renal, and kidney.

## Results

**Fig. 2:**
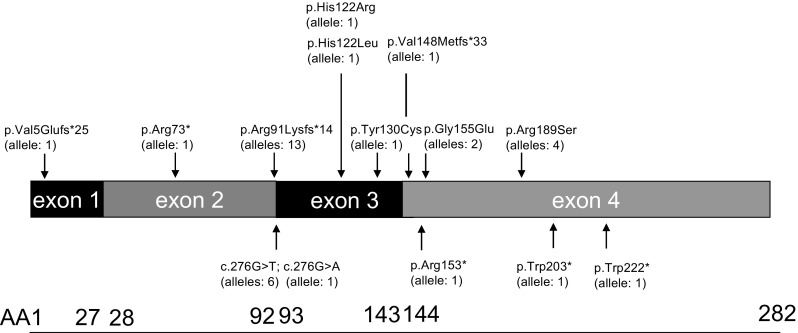
Methylmalonic aciduria and homocystinuria, cobalamine C (cblC) complementation type (*MMACHC*) mutations in patients with renal thrombotic microangiopathy (TMA). Protein prediction is depicted for all identified *MMACHC* mutations, except for the splice changes, which are indicated at the complementary DNA (cDNA) level

We identified 36 patients ([14, 21–38], including one unpublished patient) with established cblC defect and renal involvement. Diagnosis of MMACHC was confirmed biochemically in 34 patients and genetically in 18 (Table 3; for individual data, see Table S1). The most frequent *MMACHC* mutations in our cohort were c.271dupA (36 % allelic frequency), c.276G>T (17 % allelic frequency), and c.565C>A (11 % allelic frequency, Fig. 2). Most MMACHC cases were sporadic, while familial disease was noted in three sets of siblings (patient numbers 18 and, 24, 26 and 30, 27 and 28 in Table S1). Infantile onset (<12 months of age) was observed in 16 patients, and 20 patients presented with TMA beyond infancy (Table 4).

**Table 3:** Disease onset and diagnosis of cobalamin C (cblC) and complement data: findings in 36 patients with cblC defect and thrombotic microangiopathy

Variables	Findings
cblC defect	
Biochemical	94 % (*n* = 34/36)
Genetic	50 % (*n* = 18/36)
Mutations (allelic frequency)	
c.271dupA	36 % (*n* = 13/36)
c.276G>T	17 % (*n* = 6/36)
c.565C>A	11 % (*n* = 4/36)
Complement system	
Normal	87 % (*n* = 13/15)
CFH-autoantibodies	6.6 % (*n* = 1/15)
*CFH* mutation	6.6 % (*n* = 1/15)

**Table 4:** Methylmalonic aciduria and homocystinuria, cobalamin C (cblC) complementation type (MMACHC): general, renal, and extrarenal characteristics

Manifestation data	
Onset of disease	
Infantile onset	44 %, (*n* = 16/36)
Noninfantile onset	56 %, (*n* = 20/36)
Characteristics of renal disease	
Proteinuria and hematuria	100 %, (*n* = 27/27)
Hemolytic uremic syndrome	66 %, (*n* = 24/36)
Normotensive	10 %, (*n* = 2/20)
Hypertensive	90 %, (*n* = 18/20)
Nephrotic syndrome	11 %, (*n* = 3/27)
Chronic kidney disease (CKD) 1	11 %, (*n* = 4)
CKD 2–5	89 %, (*n* =32)
Dialysis	22 %, (* n* = 8)
Kidney transplant	6 %, (*n* = 2)
Extrarenal manifestations	
Neurological impairment	44 %, (*n* = 16/36)
Cardiopulmonary involvement	39 %, (*n* = 14/36)
Pulmonary hypertension	17 %, (*n* = 6/36)

**Fig. 3:**
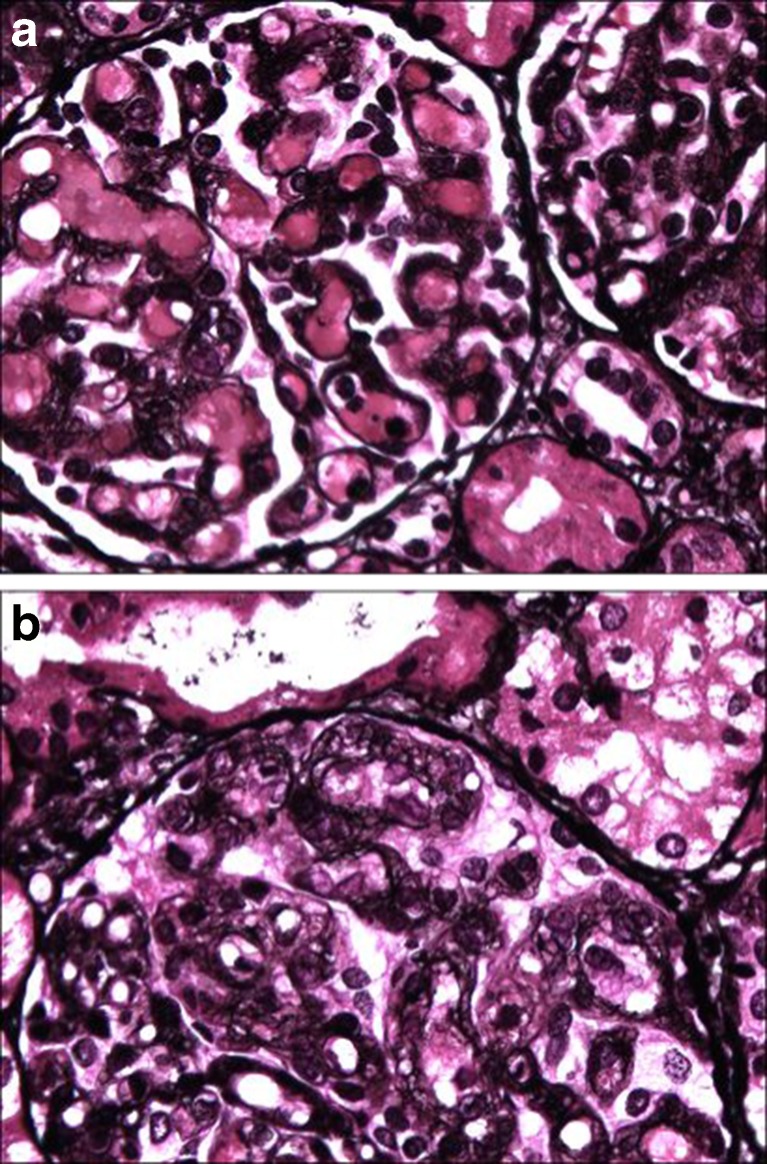
Representative renal histology in cobalamin C (clbC) defect. **a** Fibrin thrombi in the majority of glomerular capillaries (patient 18). **b** Thickening and splitting of the glomerular basement membrane (patient 18)

All patients were clinically diagnosed with TMA characterized by (microvascular thrombosis with) thrombocytopenia, hemolytic anemia, and red blood cell fragmentation [39]. Twenty-four of the 36 patients met (diarrhea-negative) hemolytic uremic syndrome (HUS) criteria defined by the classic triad of hemolytic anemia, uremia, and thrombopenia [40]. Renal biopsies or autopsies were performed in 16 patients and revealed TMA in all of them (in two patients who were initially classified as membranoproliferative glomerulonephritis, biopsy reanalysis was consistent with TMA [31]). In general, a uniform histomorphological pattern was noted (Fig. 3, Table 5): glomerular thrombi and swelling were described in 13 cases. Duplication of the glomerular basement membrane was noted in all but one patient and intra-arterial thrombi was seen in nine.

**Table 5:** Renal histology

Patient no.	1	2	9	10	16	18	20	22	24	26	27	28	25	30	31	34
Ischemia											y					
Intra-arterial thrombi	y	n	n	y	n	y	y	y	y		y	y				y
Glomerular thrombi	y			y	y	y	y	y		y	y	y	y	y	y	y
Endothelial																
Swelling		y	n		y			y	y	y	y	y	y	y	y	y
Detachment		y	n		y											
Duplication of GBM		y	n		y	y	y	y	y	y	y	y	y	y	y	y
Glomerular sclerosis		n	n		n	n	n	n	n	n	n	n	y	n	n	n

*y* yes, *n* no, *GBM* glomerular basement membrane

Complement defects were detected in two of 15 patients screened for potential atypical HUS (aHUS): patient 20 had antifactor H autoantibodies and died from pulmonary hypertension despite treatment with hydroxycobalamin. Patient 27 had a mutation in *CFH*, underwent plasmapheresis and renal replacement therapy (RRT), and responded well to treatment with hydroxycobalamin (see Tables 6 and S1). Of the 32 patients with reduced renal function, eight required dialysis and two received a kidney graft. In four patients, glomerular filtration rate was within normal limits. Hematuria and proteinuria were present in all tested patients (*n* = 27, Table 4); three of them presented with frank nephrotic syndrome [22, 30]. Arterial hypertension was reported in 18 patients. Thirty-one patients were treated with hydroxycobalamin; 17 of them improved clinically, two stabilized, and 12 died (Table 6). In 81 % of the survivors, renal function improved. In children and adolescents, glomerular filtration rate (GFR) increased from 49.5 to 89 ml/min/1.73 m^2^. Two patients were treated with cyanocobalamin (which is ineffective to overcome the metabolic block in MMACHC patients): one of them died, and no improvement of renal function was observed in the other. Three patients did not receive metabolic treatment and died.

**Table 6:** Response to metabolic and complement-targeted therapy

Response to metabolic therapy	
Clinical recovery	54 %,* n* = 17
Improvement of GFR	81 %,* n* = 13
Stable GFR	19 %,* n* = 3
Pretreatment GFR (ml/min/1.73 m^2^)	49.5* n* = 10
Posttreatment GFR (ml/min/1.73 m^2^)	89,* n* = 10
Mortality	
Overall mortality	44 % (*n* = 16/36)
Infantile onset	56 %, (*n* = 9/16)
Noninfantile onset	35 %, (*n* = 7/20)
With neurological disease	50 %, (*n* = 8/16)
With cardiopulmonary disease	79 %, (*n* = 11/14)
Untreated	100 %, (*n* = 4/4)
Complement-targeted therapy (response)	
Plasma exchange (patients 22, 24, 27, 33)	1* out of 4
Eculizumab (patients 33, 34)	No response

*GFR* glomerular filtration rate

Five patients were treated with complement-targeted therapy (Table 6), of whom, one (patient 27 with a 3254T3 C mutation in factor H) responded. In the four nonresponders, no complement defect was detected. Mortality was higher in infants (57 %) compared with later-onset disease (35 %), resulting in an overall mortality rate of 44 %. Extrarenal involvement included neurological and cardiopulmonary disease in 44 and 39 % of patients, respectively (Table 4). It is noteworthy that among the 16 nonsurvivors, 11 patients had cardiopulmonary involvement. In seven patients, pulmonary arterial hypertension was diagnosed by right ventricle catheterization and/or cardiac ultrasound; it was fatal in four.

## Discussion

### Clinical, histopathological, and genetic characteristics of renal disease in cblC defect

The key findings in the 36 patients with MMACHC and renal disease are as follows:

(1) In general, the first manifestation of renal disease occurs early in infancy, although onset can vary widely among affected individuals (median onset is 1.5 months, range 12 days to 40 years). 

(2) Overall mortality is high (44 %).

(3) Mortality is primarily the result of cardiopulmonary problems (68 %), with pulmonary hypertension identified as a specific cause in seven patients (44 %). 

(4) Renal impairment (as defined by reduced glomerular filtration) is variable and ranges from insignificant to initiation of RRT, which is necessary in 22 % of patients. 

(5) Consistent features of renal disease in all patients are intravascular hemolysis, hematuria, and proteinuria.

(6) Consistently, pathological changes predominantly show glomerular thrombotic microangiopathies [41], including glomerular fibrin thrombi, endothelial swelling, and duplication of the glomerular basement membrane. 

(7) The majority of patients respond to therapy with hydxroycobalamin. 

(8) As could be expected from its high allelic frequency, the frameshift mutation c.271dupA represented the most frequent allele in our cohort, followed by the splice-site mutation c.276G>T and the missense mutation c.565C>A. As all patients with a c.276G>T allele originated from the Dutch “Bible belt” or adjacent regions in Germany, a founder effect is highly likely here.

### Pathophysiologic considerations

Given the fact that all patients demonstrated microangiopathic hemolytic anemia [elevated lactose dehydrogenase (LDH), reduced haptoglobin levels, and/or fragmentocytes), the concept of endothelial cell damage appears to be a central pathogenic factor. This notion is clearly supported by the histological evidence of endothelial damage, i.e., endothelial swelling, detachment, and duplication of the glomerular basement membrane. The fact that the latter was observed in all but one case probably reflects the chronicity of the lesions.

The etiology of endothelial damage remains largely unknown. Homocysteine, unlike methylmalonic acid, is known to damage endothelial cells [42]. Half of the patients in this review had intermediate levels of homocysteinemia (30–100 μmol/l) at presentation; the others showed severe hyperhomocysteinemia (homocysteine levels > 100 μmol/l). Interestingly, isolated (i.e. without methylmalonic acidemia) intermediate or even severe hyperhomocysteinemia—as observed in methylenetetrahydrofolate reductase (MTHFR) deficiency (OMIM 607093) or in homocystinuria due to cystathionine beta-synthase (CBS) deficiency (OMIM 236200)—have not been reported to cause any specific renal (e.g., HUS) or cardiopulmonary (e.g., pulmonary hypertension) disease apart from thromboembolism. Possibly, additional biochemical abnormalities such as methylmalonic acidemia and/or methionine deficiency—as in cblC defect (this study) or in methylenetetrahydrofolate dehydrogenase 1 (MTHFD1) deficiency (OMIM 172460, [43, 44])—are required to develop TMA. The notion that it takes two or more toxic metabolites to trouble endothelial cells is supported by the occasional occurrence of pseudo-TMA in nutritional B_12_ deficiency [45], which—like MMACHC and MTHFD1 deficiency—is characterized by combined hyperhomocysteinemia and methylmalonic acidemia and low methionine levels. This would also hold true for such findings in some other Cbl defects, such as cblD, cblF, cblJ, and cblX variants, which are much more rare [3].

The fact that only a subgroup (minimum 36 of >500 reported patients) of patients with MMACHC are affected by TMA requires further investigation. Apart from the reduced penetrance seen in many mendelian disorders, other factors in addition to homocysteine levels could contribute to the manifestation of TMA. This fits with the observation of discordant families in a recent study reporting asymptomatic MMACHC cases (prior to treatment with hydroxylcobalamin) [46] and one from our hospital [clinically unaffected sister of patient 29 (homozygous for c.276G>T)] with comparable homocysteine levels (Kömhoff, and Berger, unpublished data). That TMA may be underreported because its features are too subtle in the presence of a predominant neurocognitive or cardiopulmonary disease or because the patient dies before renal disease develops cannot be excluded.

Comparison of the the study reported here with cohorts of children with suspected or proven aHUS/complement defects [47, 48] allows a number of interesting considerations: median age of presentation in MMACHC with renal disease (1.5 months) was much earlier than in the French ( 1.5 years, [47]) or Dutch (80 % older than 1 year of age at presentation, [48]) aHUS cohorts. Renal impairment, however, was much more pronounced in both the Dutch and French aHUS cohorts, with 44 % [48] and 59 % [47], respectively, of patients requiring RRT at presentation, compared with 22 % in MMACHC. Early presentation with relatively mild renal impairment probably reflects that MMACHC is a severe systemic disease resulting in early referral for multiple reasons when renal disease has not yet advanced. In contrast to the comparatively low mortality of 2 % [48] and 8 % [47] in the Dutch and French aHUS cohorts, respectively, overall mortality with cblC defect is high (44 %). In 11 of the 16 nonsurvivors, a cardiopulmonary problem was noted, with pulmonary arterial hypertension diagnosed in seven. The concept of cardiorenal syndrome with pulmonary hypertension and TMA in MMACHC deficiency is supported by hemolytic anemia in an infant with cblC defect who died from cor pulmonale [49] and by increased creatinine and LDH levels in another infant who died from pulmonary hypertension and MMACHC [50]. The increased risk for pulmonary hypertension should thus prompt a thorough evaluation of MMACHC patients by a pediatric cardiologist with expertise in pulmonary hypertension.

## Diagnosis

MMACHC should be considered in all children, adolescents, and young adults with unclear intravascular hemolysis, hematuria, and proteinuria, irrespective of renal and neurological impairment. Homocysteine blood concentrations > 20 μmol/l with normal renal function and >30 with renal failure require further biochemical and genetic workup [4]. It is important to realize that total Cbl levels are unrealiable indicators of a functional Cbl deficiency [51].

## Therapy

The general principles of metabolic therapy have recently been described in detail [4]. Whenever MMACHC is suspected, we consider it mandatory to promptly initiate treatment with parenteral administration of hydroxycobalamin without waiting for confirmation by genetic testing. A practical guideline is given in Table 3. It should be kept in mind, however, that some patients are difficult to treat, even with intense therapy [52]. In the event of a patient with TMA and pulmonary hypertension in whom MMACHC and a complement defect coincide (Table S1, patient 20), we strongly advocate the use of a C5 inhibitor [53] instead of plasma exchange to inhibit complement activation, as the latter carries an extremely high risk for transfusion-related acute lung injury (TRALI) [54]. When general anesthesia is required, it should be taken into account that nitrous oxide is potentially toxic to patients with MMACHC disease and needs to be avoided, because it depletes the body stores of B_12_ and inhibits methionine synthase activity [55, 56] Table 7.

**Table 7:** Metabolic therapy in patients with cobalamin C (cblC) defect (modified from [4], used with permission)

Medication	Recommended dose	Frequency	Efficacy	Target biochemical parameter
Hydroxycobalamin: i.m., i.v. or intranasally [20]	0.3 mg/kg/day	Start once daily	Established	Reducing serum MMA and tHCY levels Normalizing plasma MET and hematological parameters
Betaine oral	250 mg/kg/day	tid	Established	Reducing HCY, increasing MET
Folinic acid oral	5–15 mg/day	tid	Theoretical	Reducing HCY, increasing MET
Levocarnitine oral	50–100 mg/kg/day	tid	Theoretical	Unnecessary in case of normal MET

*HCY* homocysteine, *MET* methionine, *MMA* methylmalonic acid, *tid* three times a day

This retrospective analysis has significant limitations mainly resulting from (a) the long period (>35 years) over which patients were reported and during which significant advances have been made in the field of diagnostics and therapeutics; and (b) the heterogeneity of centers using different diagnostic and therapeutic approaches. We think, however, that the consistent features of MMACHC compensate for the heterogeneity and thereby allow us to draw the following valid conclusions:

Renal TMA due to CblC defect is a devastating disease with high mortality in which mild renal impairment may distract the nephrologist from detecting severe cardiopulmonary involvement. However, when recognized and treated at an early stage, complete clinical recovery is possible. Diagnosis is simple, and therapy is cheap and successful in the majority of patients. We therefore strongly recommend [57] sceening for MMACHC in all patients with intravascular hemolysis in combination with hematuria and proteinuria.

## Electronic supplementary material

Below is the link to the electronic supplementary material.

ESM 1 (DOC 201 kb)
