# Supplementary material for: Renal thrombotic microangiopathy in patients with cblC defect: review of an under-recognized entity
Source: Pediatr Nephrol. 2016 Jun 11;32(5):733–41. doi: 10.1007/s00467-016-3399-0 (PMC5368212; doi:10.1007/s00467-016-3399-0)
Supplement: Supplementary file 1 — (DOC 201 kb) [file 467_2016_3399_MOESM1_ESM.doc]

Table S1a: Genetic, biochemical and clinical data of 16 infants with Methylmalonic aciduria and homocystinuria, cblC type

| pat | age | diagnosis | type of mutation | type of  TMA | RR | Creat/BUN | RRT | urine | N | CP | therapy | outcome  survival | GFR | ref |
| --- | --- | --- | --- | --- | --- | --- | --- | --- | --- | --- | --- | --- | --- | --- |
| 1 | neo | biochemical | ND | HUS |  | ↑ |  |  |  |  | HyC | **†**, 40 d |  | [14] |
| 2 | neo | biochemical | ND | HUS |  | ↑ |  |  |  | resp. fail. | HyC | **†**, 52 d |  | [58] |
| 3 | 12d | biochemical | ND | HUS | ↑ | ↑ |  | H, P | y |  | HyC, F, B, | rec., GFR ↑ | 12 - nl. | [29] |
| 4 | 14 d | biochemical | ND | HUS |  | ↑ |  | H, P | y | resp. fail. | HyC, F, B | **†**, 44 d |  | [58] |
| 5 | 15 d | c.271dupA c.666C>A | fs  stop | TMA | ↔ | ↔ |  | H, P | y |  | HyC, F, B | rec., | 107- nl. | [27] |
| 6 | 21 d | c.271dupA  c.271dupA | fs  stop | HUS |  | ↑ |  | H, P | y |  | HyC, F, B | rec., GFR ↑ | 29 - nl. | [27] |
| 7 | 25 d | biochemical | ND | HUS | ↑ | ↑ |  | H, P | y |  | HyC, F, B | rec., GFR ↑ | 14 - 117 | [32] |
| 8 | 28 d | c.271dupA  c.271dupA | fs  fs | TMA |  | ↔ |  | H, P | y | left card. fail. | HyC, F, B | rec., | 45 - nl. | [27] |
| 9 | 28d | biochemical | ND | TMA | ↑ | ↑ |  |  | y |  | HyC, B | **†** | 33 - 12 | [34] |
| 10 | 29 d | biochemical | ND | HUS |  | ↑ |  |  | y |  | HyC | **†** 35 d |  | [14] |
| 11 | 31 d | c.271dupA  c.271dupA | fs  fs | HUS |  | ↑ |  | H, P | y |  | HyC, F, B | rec., GFR ↑ | 35 - nl. | [27] |
| 12 | 1 m | biochemical | ND | HUS |  | ↑ |  |  | n | resp. fail. |  | **†** |  | [58] |
| 13 | 32d | biochemical | ND | HUS | ↑ | ↑ |  | H, P | y | resp. fail. | HyC | **†** | 26 - 17 | [33] |
| 14 | 40 d | biochemical | ND | HUS |  | ↑ |  | H, P | y | resp. fail. | HyC | **†**, 90 d |  | [58] |
| 15 | 44 d | biochemical | ND | HUS |  | ↑ |  |  | y |  | HyC | stable |  | [23] |
| 16 | 3 m | biochemical | ND | HUS | ↑ | ↑ |  | H, P | n | cardiac fail. | CyC | **†**, at 3 m |  | [19] |

Table S1b: : Genetic, biochemical and clinical data of 20 subjects diagnosed beyond infancy with Methylmalonic aciduria and homocystinuria, cblC type

| pat | age | diagnosis | type of mutation | def | RR | Creat/BUN | RRT | urine | compl. | N | CP | therapy | outcome  survival | GFR | ref |
| --- | --- | --- | --- | --- | --- | --- | --- | --- | --- | --- | --- | --- | --- | --- | --- |
| 17 | >1 y | c.271dupA/ c.565C>A | fs  missense | HUS |  | ↑ | y |  |  | ? |  | HyC | KTX 9 y |  | [36] |
| 18 | 1.5y | c.276G>T/ c.271dupA | splice  fs | TMA | n | ↑ |  | H, P | normal | yes | PH |  | **†**, 1.5 y | 117 - 19 | [26] |
| 19 | 1.5 y | c.609G>A/ c.217C>T | missense/  stop | HUS | ↑ | ↑ |  | H, P |  | no |  | HyC, F, B | rec., GFR ↑ | 45 - 112 | [21] |
| 20 | 2.5 y | c.464G.A.  c.464G.A | missense  missense | HUS | ↑ | ↑ | y | H, P | aFH |  | PH | HyC | **†**, 2.5 y | 31 - 10 | [26] |
| 21 | 2.7y | biochemical | ND | HUS | ↑ | ↑ |  | H, P |  | yes | MoF | HyC, F, B | GFR ↑, **†** | 17 66 | [21] |
| 22 | 3 y | c.276G>T/  c.442_444delinsA | splice  fs | HUS | ↑ | ↑ | Y | H, P | normal* |  | PH | PE | KTX,**†**, 10 y | 98 – 80 | [26, 28, 59] |
| 23 | 3.3 y | c.609G>A  c.365A>T | missense  missense | HUS | ↑ | ↑ |  | H, P |  | no |  | HyC, F, B | rec., GFR ↑ | 28 - 119 | [21] |
| 24 | 4 y | c.276G>T/ c.271dupA | splice  fs | TMA | ↑ | ↑ |  | H, P | Normal | No | PH | PE, HyC, F, B6, B | rec., GFR ↑ | 130 - 120 | [26] |
| 25 | 4 y | c.276G>T/ c.271dupA | splice  fs | TMA | ↑ | ↔ |  | H,NS | normal | no |  | HyC, F, B6, B | rec., | nl. | [22] |
| 26 | 4 y | biochemical | ND | HUS | ↑ | ↑ |  | H, P | normal | no |  | HyC, F, B, C | rec., GFR ↑ | 53 - 97 | [31] |
| 27 | 6 y | biochemical | ND | TMA | ↑ | ↑ | y | H,NS | CFH | no |  | PE, HyC, F, B | off dialysis | 19 - 99 | [30] |
| 28 | 8 y | biochemical | ND | TMA | ↑ | ↔ |  | H,NS | normal | no |  | HyC, F, B | rec., | 119 - 108 | [30] |
| 29 | 8 y | c.276G>T  c.276G>T | splice  splice | TMA | ↑ | ↑ |  | H, P | normal | no | PH | HyC, F, B, B6 | **†**, 9 y | 90 -70 | * |
| 30 | 12 y | biochemical | ND | HUS | ↑ | ↑ |  | H, P | normal | no |  | HyC, F, B, C | GFR ↑ | 49-78 | [31] |
| 31 | 14 y | c.276G>A  c.14_24del11 | splice  fs | TMA | ↑ | ↑ |  | H, P | normal | no | PH | CyC | stabilized | 84 - 80 | [26, 60] |
| 32 | 16 y | biochemical | ND | TMA | ↑ | ↑ |  | H, P | normal | no |  | HyC, F, B | **†** | 22- 30 | [38] |
| 33 | 18 y | c.271dupA c.565C>A | fs  missense | HUS | ↑ | ↑ | y | H, P | normal | yes |  | PE, Ec, HyC, F, | off dialysis | 14 -61 | [24] |
| 34 | 20 y | c.271dupA c.389A>G | fs  missense | HUS | ↑ | ↑ | y | H, P | normal | no |  | Ec, HyC, | off dialysis | 21-31 | [25] |
| 35 | 23 y | c.565C>A  c.565C>A | missense  missense | HUS | ↑ | ↑ | y | H, P | normal | no |  | HyC | ESRD |  | [35] |
| 36 | 40 y | c.457C>T  c.365A>G | stop  missense | TMA |  |  |  |  |  | yes |  | HyC | **†** |  | [37] |

**Abbreviations:** †: deceased,*: Kömhoff and Berger, unpublished observation. Creat: creatinine, BUN: blood urea nitrogen, RRT: renal replacement therapy, H, P: hematuria & proteinuria, NS: nephrotic syndrome, compl: complement; therapy: HyC, hydroxycobalamin, F; folate, B: betain, B6: vitamin B6, C: carnitine, organ: organ involvement, N: neurology, G: gastritis, E: eyes, fail: failure, PH: pulmonary hypertension, MOF: multi organ failure, KTX: renal transplant, neo: neonatal, y: years, ND: no data

**References**

58. Russo P, Doyon J, Sonsino E, Ogier H, Saudubray JM (1992) A congenital anomaly of vitamin B12 metabolism: a study of three cases. Hum Pathol 23:504-512

59. Davin JC, Buter N, Groothoff J, van Wijk J, Bouts A, Strain L, Goodship T (2009) Prophylactic plasma exchange in CD46-associated atypical haemolytic uremic syndrome. Pediatr Nephrol 24:1757-1760

60. Losito A, Pittavini L, Covarelli C (2012) Thrombotic microangiopathic nephropathy, pulmonary hypertension and nephromegaly: case report of a patient treated with endothelin receptor antagonist. Clin Nephrol 77:164-170
